# Complete Genome Sequence of *Mannheimia haemolytica* Strain 42548 from a Case of Bovine Respiratory Disease

**DOI:** 10.1128/genomeA.00318-13

**Published:** 2013-05-30

**Authors:** Christopher Eidam, Anja Poehlein, Geovana Brenner Michael, Kristina Kadlec, Heiko Liesegang, Elzbieta Brzuszkiewicz, Rolf Daniel, Michael T. Sweeney, Robert W. Murray, Jeffrey L. Watts, Stefan Schwarz

**Affiliations:** Institute of Farm Animal Genetics, Friedrich-Loeffler-Institut (FLI), Neustadt-Mariensee, Germanya; Department of Genomic and Applied Microbiology, Goettingen Genomics Laboratory, Institute of Microbiology and Genetics, Georg-August-University of Goettingen, Goettingen, Germanyb; Zoetis, Kalamazoo, Michigan, USAc

## Abstract

*Mannheimia haemolytica* is the major bacterial component in the bovine respiratory disease complex, which accounts for considerable economic losses to the cattle industry worldwide. The complete genome sequence of *M. haemolytica* strain 42548 was determined. It has a size of 2.73 Mb and contains 2,888 genes, including several antibiotic resistance genes.

## GENOME ANNOUNCEMENT

The gammaproteobacterium *Mannheimia haemolytica* is a facultatively pathogenic, Gram-negative bacterium of the upper respiratory tract and nasopharynx of ruminants. It is considered the major bacterial agent of the bovine respiratory disease (BRD) complex. BRD accounts for over $3 billion in losses to the cattle industry every year (1). Viral pathogens are believed to initiate the disease and pave the way for secondary bacterial infections. While vaccines are used to prevent the onset of BRD, antimicrobial agents are also used, especially in cases when *M. haemolytica*, *Pasteurella multocida*, or *Histophilus somni* is involved (2). The development of antimicrobial resistance among bacterial BRD pathogens is of growing concern (3), as antimicrobial multiresistance conferred by the integrative and conjugative element ICE*Pmu1* has recently been described (4, 5). ICE*Pmu1* carries 12 antimicrobial resistance genes, some of which also confer resistance against gamithromycin and tildipirosin, the latest antimicrobial agents approved for food-animal use (6). The complete genome sequence of the multiresistant *M. haemolytica* isolate 42548 will further our understanding of genome structure and function as well as provide insight into the transfer, acquisition, and genetic basis of antimicrobial multiresistance.

Genomic DNA was isolated from *M. haemolytica* 42548 using the MasterPure Complete DNA and RNA Purification kit (Epicenter, Madison, WI). Whole-genome sequencing was performed using the 454 GS-FLX titanium XL system (titanium GS70 chemistry; Roche Life Science, Mannheim, Germany) and the Genome Analyzer IIx (Illumina, San Diego). We performed 454 shotgun sequencing, which produced 135,530 single-end reads with an average length of 612 bases (28.6× coverage), while Illumina sequencing resulted in 3,696,221 of 112-bp paired-end reads (116× coverage). The initial hybrid *de novo* assembly performed using the MIRA software resulted in 316 contigs. Gaps between contigs were closed using PCR amplification, subsequent Sanger sequencing (7), BigDye 3.0 chemistry, and an ABI3730XL capillary sequencer (Applied Biosystems, Life Technologies GmbH, Darmstadt, Germany). Gap closure was done using the Gap4 (v.4.11) software (8). The genome of *M. haemolytica* 42548 consists of a single chromosome of 2.73 Mb with a GC content of 41.05%. The initial gene prediction was done using YACOP (9) and Glimmer (10), while rRNA and tRNA were identified with RNAmmer and tRNAscan (11, 12). This revealed a total number of 2,888 genes, including 6 rRNA gene clusters and 61 tRNA genes. Protein genes were annotated using Swiss-Prot and TrEMBL (13), followed by manual curation based on comparison with genes predicted by IMG/ER (14). This resulted in 2,807 protein-encoding genes with assigned functions. Additionally, 55 putative transposases and one CRISPR region of the I-C/Dvulg subtype were detected (MHH_c16440-16500) (15). Several antibiotic resistance genes were located within a putative integrative and conjugative element, tentatively designated ICE*Mh1*, which shared high similarities with ICE*Pmu1*. ICE*Mh1* carried the resistance genes *aphA1* (MHH_c22550), *strA* (MHH_c22570), *strB* (MHH_c22560), *sul2* (MHH_c22580), and *tetR*-*tet*(H) (MHH_c23150-23160), which accounted for the multiresistance phenotype.

### Nucleotide sequence accession number.

The genome sequence of *M. haemolytica* 42548 has been deposited at DDBJ/EMBL/GenBank under the accession no. CP005383.
